# Duration of Contagion of Foot-And-Mouth Disease Virus in Infected Live Pigs and Carcasses

**DOI:** 10.3389/fvets.2020.00334

**Published:** 2020-06-11

**Authors:** Carolina Stenfeldt, Miranda R. Bertram, George R. Smoliga, Ethan J. Hartwig, Amy H. Delgado, Jonathan Arzt

**Affiliations:** ^1^Foreign Animal Disease Research Unit, Plum Island Animal Disease Center, Agricultural Research Service, United States Department of Agriculture, Greenport, NY, United States; ^2^Department of Diagnostic Medicine/Pathobiology, Kansas State University, Manhattan, KS, United States; ^3^PIADC Research Participation Program, Oak Ridge Institute for Science and Education, Oak Ridge, TN, United States; ^4^Monitoring and Modeling, Center for Epidemiology and Animal Health, Animal and Plant Health Inspection Service, United States Department of Agriculture, Fort Collins, CO, United States

**Keywords:** foot-and-mouth disease, foot-and-mouth disease virus, FMD, FMDV, pig, transmission, contagion

## Abstract

Data-driven modeling of incursions of high-consequence, transboundary pathogens of animals is a critical component of veterinary preparedness. However, simplifying assumptions and excessive use of proxy measures to compensate for gaps in available data may compromise modeled outcomes. The current investigation was prospectively designed to address two major gaps in current knowledge of foot-and-mouth disease virus (FMDV) pathogenesis in pigs: the end (duration) of the infectious period and the viability of FMDV in decaying carcasses. By serial exposure of sentinel groups of pigs to the same group of donor pigs infected by FMDV A24 Cruzeiro, it was demonstrated that infected pigs transmitted disease at 10 days post infection (dpi), but not at 15 dpi. Assuming a latent period of 1 day, this would result in a conservative estimate of an infectious duration of 9 days, which is considerably longer than suggested by a previous report from an experiment performed in cattle. Airborne contagion was diminished within two days of removal of infected pigs from isolation rooms. FMDV in muscle was inactivated within 7 days in carcasses stored at 4^o^C. By contrast, FMDV infectivity in vesicle epithelium harvested from intact carcasses stored under similar conditions remained remarkably high until the study termination at 11 weeks post mortem. The output from this study consists of experimentally determined data on contagion associated with FMDV-infected pigs. This information may be utilized to update parameterization of models used for foot-and-mouth disease outbreak simulations involving areas of substantial pig production.

## Introduction

Foot-and-mouth disease (FMD) is a high-impact viral disease capable of infecting all cloven-hoofed domestic livestock and numerous wildlife species (1). The causative agent, foot-and-mouth disease virus (FMDV; genus: *Aphthovirus*, family: *Picornaviridae*) causes initial infection via the upper respiratory- or gastrointestinal tracts (depending on host species), followed by systemic generalization with lameness, inappentence, and vesicular lesions on the feet and in the mouth as characteristic clinical findings (2, 3). FMD is endemic in large parts of the world, including most of Africa, the Middle East, and Asia, as well as certain regions of South America (4). By contrast, Europe, North America, Australia, and New Zealand, are kept free of FMD by means of strict regulation of import of animals and animal products as well as by extensive control programs designed to rapidly detect potential incursions. Due to the potentially catastrophic consequences associated with incursions of FMDV into previously free countries (5), substantial efforts are invested into veterinary preparedness and contingency planning. Such endeavors often include mathematical modeling of FMD outbreaks within defined geographic regions for the purpose of estimating disease spread and impact, and to evaluate the effect of applied control measures (6). Outbreak simulations are critically dependent upon appropriate parameterization of models. Essential input parameters need to reflect the detailed structure of the susceptible population, as well as intrinsic factors of host-pathogen interactions that capture the progression of infection and contagiousness within individual animals.

Model parameterization for FMD outbreak simulations is often based upon meta analyses of published experimental studies (7–9). Although there are clear benefits of utilizing available published data for this purpose, simplifying assumptions and extensive use of proxy values are often needed to compensate for gaps in available data. Specifically, the timing of infectiousness in relation to initial exposure and the appearance of clinical signs of disease is of critical value for modeling within-herd transmission of FMD. However, infectiousness, i.e., the ability of an infected animal to transmit infection to in-contact animals, is rarely directly quantified in controlled FMDV experiments. Instead, proxies such as detection of viral genome in secretions, or observed clinical signs of disease are often used to estimate infectiousness for model parameterization (7, 8).

Previous works have demonstrated that the selection and definition of proxies can lead to greatly varying estimates for the onset and duration of infectiousness in FMDV-infected cattle and pigs (10, 11). Such variation in input parameters can lead to substantially different estimates of outbreak dispersal and duration in downstream modeling. Additionally, it was demonstrated that failure to account for preclinical (incubation phase) infectiousness in FMDV-infected pigs substantially under-estimated the outcomes of FMD outbreak simulations in areas of dense pig production (10). However, a factor that has received less attention, but that is potentially of similarly critical importance for FMD modeling, is the end of infectiousness in infected hosts, which contributes to definition of the total infectious period. This factor would likely be of greatest impact in scenarios in which disease detection is delayed, or when timely depopulation of infected farms may not be possible. The latter of those circumstances will likely be an issue if an FMD incursion were to affect multiple large livestock holdings, as available resources for depopulation and carcass disposal may be overwhelmed (12–14).

Unless properly disposed of, carcasses of infected animals on depopulated farms may represent a substantial source of contagion (15). It has been reported that FMDV in carcasses will become inactivated due to post mortem acidification (16, 17). Although this has proven to be the case in muscle (18), the greatest viral loads during clinical FMD are in vesicular epithelium at peripheral sites (19). These sites are less likely to be affected by systemic acidification and core temperature changes due to autolysis and are by their peripheral location at the exterior of the carcass also in direct contact with the environment.

This current study reports the descriptive results from an experiment that was prospectively designed to evaluate the end of infectiousness in group-housed FMDV-infected pigs. Additional output includes longitudinal sampling of muscle and vesicle epithelium from carcasses of pigs that had been euthanized during the clinical phase of FMD and air sampling in rooms from which infected pigs had been removed.

## Methods

### Virus

The virus used for the current study was a cattle-derived isolate of FMDV A24 Cruzeiro that had been passaged once in pigs. The pathogenesis and transmission characteristics of this specific virus isolate in pigs have been described in detail in previous publications (19–21).

### Animals

Animal experiments were carried out within BSL3Ag research facilities at the Plum Island Animal Disease Center, New York. All experimental procedures were approved by the institutional animal care and use committee that functions to ensure ethical and humane treatment of animals (protocol 231-17). The animals used were female Yorkshire pigs weighing ~25 kg at delivery. The pigs were delivered to the facility as one batch and allowed two weeks of acclimation in the facility prior to start of the study.

### Study Design

Sixteen pigs were divided into four groups of four pigs each. Group 1, which were assigned as the “donor pigs”, were infected with FMDV by intra-oropharyngeal (IOP) inoculation as previously described (22). In brief; sedated pigs were placed in dorsal recumbency and 2ml of diluted inoculum was deposited onto the surface of the tonsil of the soft palate using a blunt-ended cannula. Each pig received a challenge dose of 100, 50% infectious doses titrated *in vivo* in pig heel bulb epithelium (23), which corresponded to 10^7^, 50% tissue culture infectious doses (TCID_50_) when back-titrations were performed using the highly sensitive LFBKαvβ6 cell line (24, 25). At 3 pre-determined time points, corresponding to 5, 10, and 15 days post inoculation (dpi) of the donor pigs, the four donor pigs were relocated to co-habitate with 4 pigs of study groups 2, 3, and 4, respectively, for 24 h each. The contact exposure consisted of co-housing the 4 donor pigs with the 4 susceptible recipients within separate animal isolation rooms with 17.5 m^2^ (group 2) or 10.2 m^2^ (groups 3 and 4) available floor area. Feed was provided on the floor at the start of the contact exposure period. After each of the 24 h exposure periods, the donor pigs were moved back to their original room and remained in isolation until the subsequent exposure period. After completion of exposure of study group 4 (16 dpi of the donors) the donor pigs were euthanized and removed from the study. The contact-exposed pigs in groups 2–4 were monitored for 14 days post exposure (dpe), or until development of fulminant FMD.

#### Sample Collection

Antemortem samples consisted of whole blood obtained by jugular venipuncture, and oropharyngeal fluid (OPF) obtained by swabbing the oropharynx using a large cotton swab. Swabs were immediately submerged in 2 ml of minimum essential media with 25 mM HEPES and were subsequently centrifuged to extract absorbed fluid. Blood samples were separated by centrifugation and aliquots of serum and OPF were stored at −70^o^C until further processing. Samples were collected prior to inoculation or exposure (0 dpi/dpe). The donor pigs were sampled every other day from 0 to 6 dpi, and again at 10 and 15 dpi. Contact-exposed pigs were sampled every other day from 0 to 10 dpe, and again at 14 dpe. Additional OPF swabs were collected so that samples were obtained from all pigs at the beginning and end of the contact exposure periods (i.e., from donor pigs, additional OPF samplings were done at 5, 11, and 16 dpi, and for contact-exposed pigs at 1 dpe).

The onset and progression of the pigs' clinical status (lesion distribution) was quantitated using a previously described scoring system (26). In brief, each of 16 digits having a characteristic FMDV lesion contributed one point toward a cumulative score, with four additional single points added for lesions within the oral cavity, on the snout, on the lower lip, and on carpal/tarsal skin, respectively, resulting in a maximum score of 20.

#### FMDV Recovery From Muscle and Vesicular Lesions Post Mortem

The viability of FMDV in muscle and vesicle epithelium after death of the animals was evaluated by post-mortem sampling at repeated time points. Initially, the four pigs from study group 2 of the transmission study reported herein were utilized for this purpose. Based on the findings from these initial samples, additional pig carcasses were recruited from unrelated experimental studies in order to extend the post mortem sampling period and to include a second strain of FMDV. These studies comprised pigs that had been infected with the same strain of FMDV A24 as was used in the transmission study, or with a bovine-derived strain of FMDV O/SKR/2010 (27). All pigs utilized for post-mortem sampling were euthanized during acute FMD by intravenous injection of an overdose of sodium pentobarbital “Fatal plus®” (85.8 mg/kg) under deep sedation (Telazol, Ketamine, and Xylazine at 3, 8, and 4 mg/kg, respectively). Carcasses were stored intact in metal cans lined with plastic bags at 4^o^C throughout the sampling periods.

From the first batch of FMDV A24-infected pigs (study group 2), samples of vesicular epithelium and skeletal muscle (semitendinosus) were obtained at 0, 1, 3, and 5 days post mortem (dpm). The second batch of FMDV A24-infected pigs were subjected to sampling of vesicular epithelium at 5, 10, and 19 dpm. The third batch of pigs were infected with FMDV O/SKR/2010, and samples of vesicle epithelium were harvested at approximately weekly intervals from 4 to 37 dpm. The fourth batch of pigs were infected with FMDV A24; muscle samples were obtained at 0, 7, and 14 dpm, with vesicle epithelium samples harvested at approximately weekly intervals from 0 to 77 dpm. The final sampling at 77 dpm also included harvest of submandibular lymph nodes, neck skin, and bone marrow.

FMDV recovery in tissue samples obtained from pig carcasses was evaluated by qRT-PCR and virus isolation (VI). Additionally, virus titrations were performed on all vesicle epithelium samples from batch 4 carcasses and on additional tissues harvested at 77 dpm that were VI-positive. Titer values expressed as log10 TCID_50_ were used for statistical analysis of virus decay: a simple linear regression with day as the independent variable and titer as the dependent variable was used to estimate the titer half-life and duration of detection.

#### Air Sampling

Air sampling in the rooms housing experimental groups 2 and 3 was performed using a Model 1,000 air pump developed by the Program Executive Office for Chemical Biological Defense (PEO-CBD), fitted with an original DFU filter assembly holding two separate Lockheed Martin polyester filter disk (1.0 lm filter, diameter 47 mm, Catalog number DFU-P-24; Lockheed Martin, Washington DC, USA) as previously described (21). The airflow through the unit was 15 l/min, and the pump was placed out of reach of the animals. The filter disk were removed and replaced at 24 h intervals. The pump was left running in between daily sample collections, and the filters were removed prior to cleaning of the animal rooms, with the pump turned off as the room was cleaned to avoid sampling of artifactually re-suspended aerosols. Pumps were started the day before the start of contact exposure, and the first filters (0 dpe) were removed before the FMDV-infected donor pigs entered the room. Air sampling in room 2 continued through 8 days after euthanasia of the contact-exposed pigs and washing of the room. Air sampling in room 3 continued for 24 h after removal of the last pigs.

### FMDV RNA Detection in Serum, Swabs and Tissues

Tissue samples (ca 20 mg) were thawed and macerated using a TissueLyser bead beater (Qiagen, Valencia, CA) and stainless steel beads (Qiagen cat. no. 69989). Air filters were cut in quarters and disrupted using a similar approach as for tissue samples, but with washed glass beads (combination of bead sizes 425–600 μm and ≤106 μm; Sigma cat. nos. G4949/G8772). The air filter “homogenate” was subsequently centrifuged to extract the fluid that had been absorbed by the filter. Tissue- and air filer macerates, serum, and swab samples were analyzed using quantitative real-time RT-PCR (qRT-PCR), targeting the 3D region of the FMDV genome (28) with forward and reverse primers adapted from Rasmussen et al. (29), and chemistry and cycling conditions as previously described (30). Cycle threshold values were converted into FMDV genome copy numbers (GCN) per ml by use of a standard curve derived from analysis of 10-fold dilutions of *in-vitro* synthesized FMDV RNA. The equation of the curve of GCN vs. Ct values was further adjusted for dilutions used during processing of samples.

### Virus Isolation

Aliquots of macerated tissue samples and air filters were cleared from debris and potential bacterial contamination by centrifugation through Spin-X® filter columns (Costar Cat.no 8163). Clarified samples were subsequently analyzed for infectious FMDV through virus isolation (VI) on LFBK αvβ6 cells (24, 31), following a protocol previously described (32). Presence of FMDV was further confirmed by qRT-PCR analysis of VI cell culture supernatants.

OPF samples obtained from donor pigs at the beginning of each of the 3 contact exposure periods, as well as select post mortem tissue samples (see above), were titrated on LFBK αvβ6 cells in micro-titer plates using similar methods as described above, but with end point titers (TCID_50_) determined using standard methods (33).

## Results

### Donor Pigs

**Figure 1:**
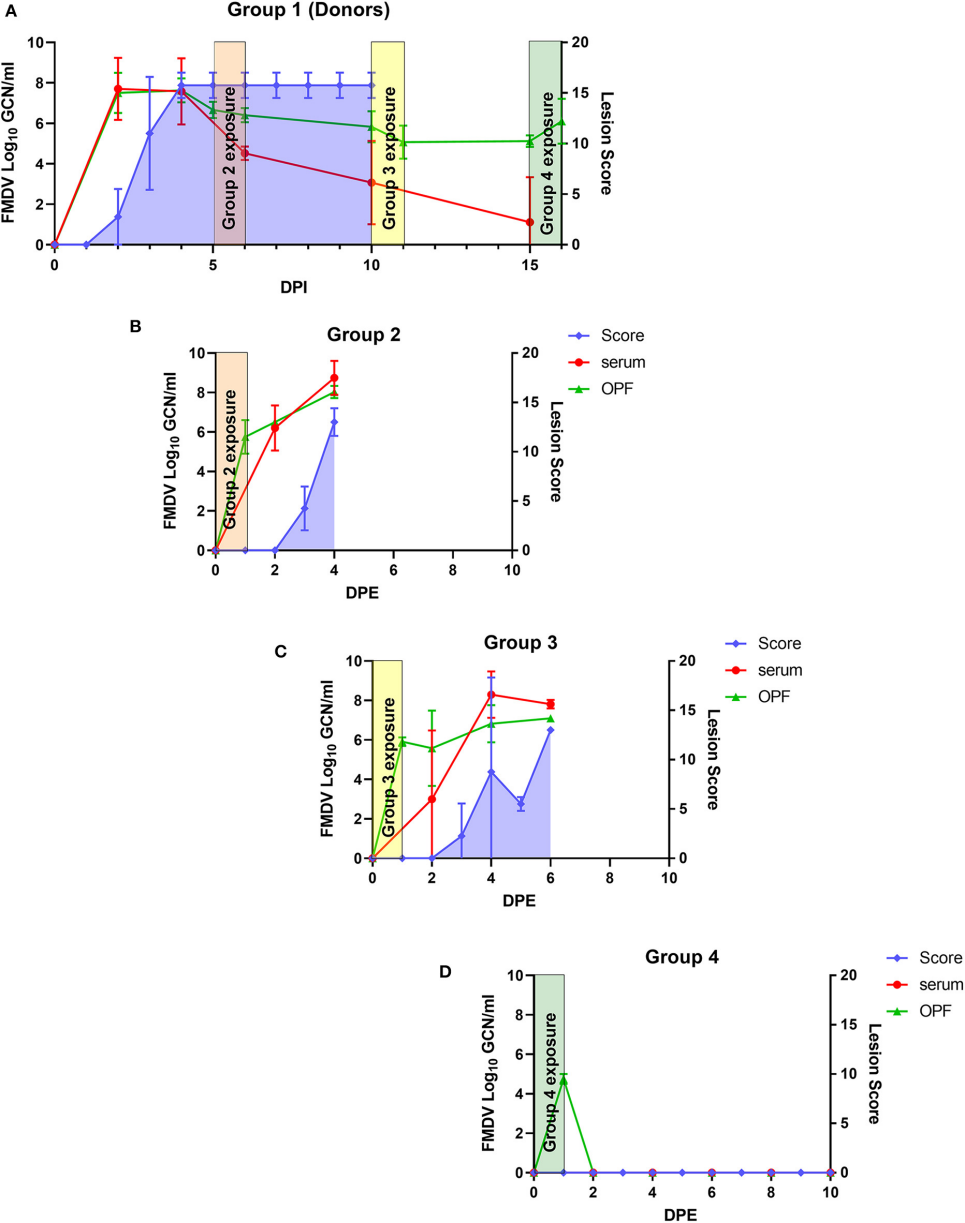
Transmission dynamics of FMDV during late infection. **(A)** Four pigs in group 1 (donors) were infected with FMDV A24 Cruzeiro by intra-oropharyngeal inoculation on day 0. **(B–D)** The 4 donor pigs were subsequently co-mingled with study groups 2, 3, and 4, for 24 h each, on days 5, 10, and 15 post infection, respectively. Graphs show average quantities (geometric means +/- standard deviation) of FMDV RNA (log_10_ GCN/ml) in serum (red) and oropharyngeal fluid (OPF; green), as well as cumulative lesion scores (shaded blue). Orange, yellow, and green rectangles correspond to the time frame during which the donors were housed together with groups 2, 3, and 4, respectively.

All 4 pigs within the donor group developed clinical FMD of a similar severity and timeline as has previously been described for this virus isolate (19, 22). Vesicular lesions appeared at 2–3 dpi, and all pigs were viremic and shedding high quantities of FMDV RNA (6.15–8.25 log_10_ GCN/ml) by the first sampling time point at 2 dpi (Figure 1, Table 1). All four donor pigs had reached their maximum cumulative lesion scores by 4 dpi (Figure 1).

**Table 1:** Donor pig characteristics during contact exposure periods.

**Experimental group**	**Contact exposure time point (dpi of donors at start of exposure)**	**Mean FMDV RNA in OPF at start of exposure (log_**10**_ GCN/ml)**	**Mean FMDV RNA in OPF at end of exposure (log_**10**_ GCN/ml)**	**Number of titer-positive OPF samples at start of exposure**	**Highest FMDV titer in OPF at start of exposure (log_**10**_ TCID_**50**_/ml)**	**Confirmed transmission to contact-exposed pigs**
2	5	6.66	6.41	3	3.250	Yes
3	10	5.82	5.07	1	2.625	Yes
4	15	5.12	6.11	0	NA	No

### Contact Transmission of FMD

#### Group 2; 5 dpi

At the beginning of the first contact exposure at 5 dpi, the clinical conditions of the donor pigs were improving. They were all non-febrile and ambulant, but with moderate lameness and with ruptured vesicular lesions on their feet. The range of FMDV RNA detected in OPF from the donor pigs at 5 dpi was 6.12–7.08 log_10_ GCN/ml (average 6.66 log_10_ GCN/ml). Three of four donor pigs had measurable FMDV titers in OPF at 5 dpi, ranging from 10^2.25^ to 10^3.25^ TCID_50_ (Table 1). At 6 dpi, corresponding to the end of contact exposure of group 2, the level of FMDV RNA detection in OPF of the donors was 6.08–6.89 log_10_ GCN/ml (average 6.41 log_10_ GCN/ml), and none of the OPF samples had a measurable FMDV titer.

The contact-exposed pigs in group 2, which were exposed to the donors from 5 to 6 dpi, all had moderate levels of FMDV RNA in OPF swabs at the end of the contact exposure (1 dpe; range 5.03-6.85 log_10_ GCN/ml, average 5.75 log_10_ GCN/ml). All 4 pigs in group 2 were viremic at 2 dpe, and all had vesicular lesions at 3 dpe (Figure 1B). The pigs in group 2 were euthanized at 4 dpe and were subsequently subjected to post mortem sampling of muscle and vesicular lesions.

#### Group 3; 10 dpi

At 10 dpi, corresponding to the start of contact exposure of group 3, the donor pigs had recovered from apparent clinical FMD. They were all ambulatory and moving freely without marked lameness. Vesicular lesions were healing, although re-epithelializing vesicular erosions were present on their feet. At 10 dpi, FMDV RNA shedding in OPF of the donor pigs ranged from 4.79 to 6.63 log_10_ GCN/ml (average 5.82 log_10_ GCN/ml). An FMDV titer of 10^2.62^TCID_50_ was measured in 1 of 4 OPF samples obtained at that time point, whereas the OPF of the 3 additional pigs was negative for infectious virus. At the end of exposure of group 3, at 11 dpi, the measured FMDV RNA detection in OPF of the donor pigs was 4.05–6.05 log_10_ GCN/ml (average 5.07 log_10_ GCN/ml; Table 1).

FMDV RNA was present in OPF swabs of all the contact-exposed pigs of group 3 at the end of exposure (1 dpe; range 5.64–6.85 log_10_ GCN/ml, average 5.91 log_10_ GCN/ml). Two of the pigs were viremic at 2 dpe, with viremia confirmed in the remaining two pigs at the subsequent sampling at 4 dpe. Two of the pigs had vesicular lesions at 3 dpe, and vesicles were detected at 4 and 5 dpe, respectively, in the remaining 2 pigs (Figure 1C). The two pigs that developed vesicles at 2 dpe were euthanized on the subsequent day, and the remaining two pigs were both euthanized at 6 dpe.

#### Group 4; 15 dpi

At 15 dpi, which was the start of contact exposure of group 4, the clinical conditions of the donor pigs had further improved. All 4 pigs still had recognizable healing FMD lesions on their feet and were shedding detectable quantities of FMDV RNA in OPF (range 4.68–5.31 log_10_ GCN/ml, average 5.12 log_10_ GCN/ml). At the end of the final contact exposure, FMDV RNA levels in OPF from the donor pigs were 4.85–6.28 log_10_ GCN/ml (average 6.11 log_10_ GCN/ml). All virus titrations of OPF obtained from the donor pigs at 15 dpi were negative (Table 1).

FMDV RNA was detectable in OPF from all contact-exposed pigs of group 4 at the end of exposure (range 4.49–5.09 log_10_ GCN/ml, average 4.76 log_10_ GCN/ml). However, all subsequent samples were FMDV-negative, and none of the 4 pigs developed clinical FMD (Figure 1D).

### Detection of FMDV in Air

**Figure 2:**
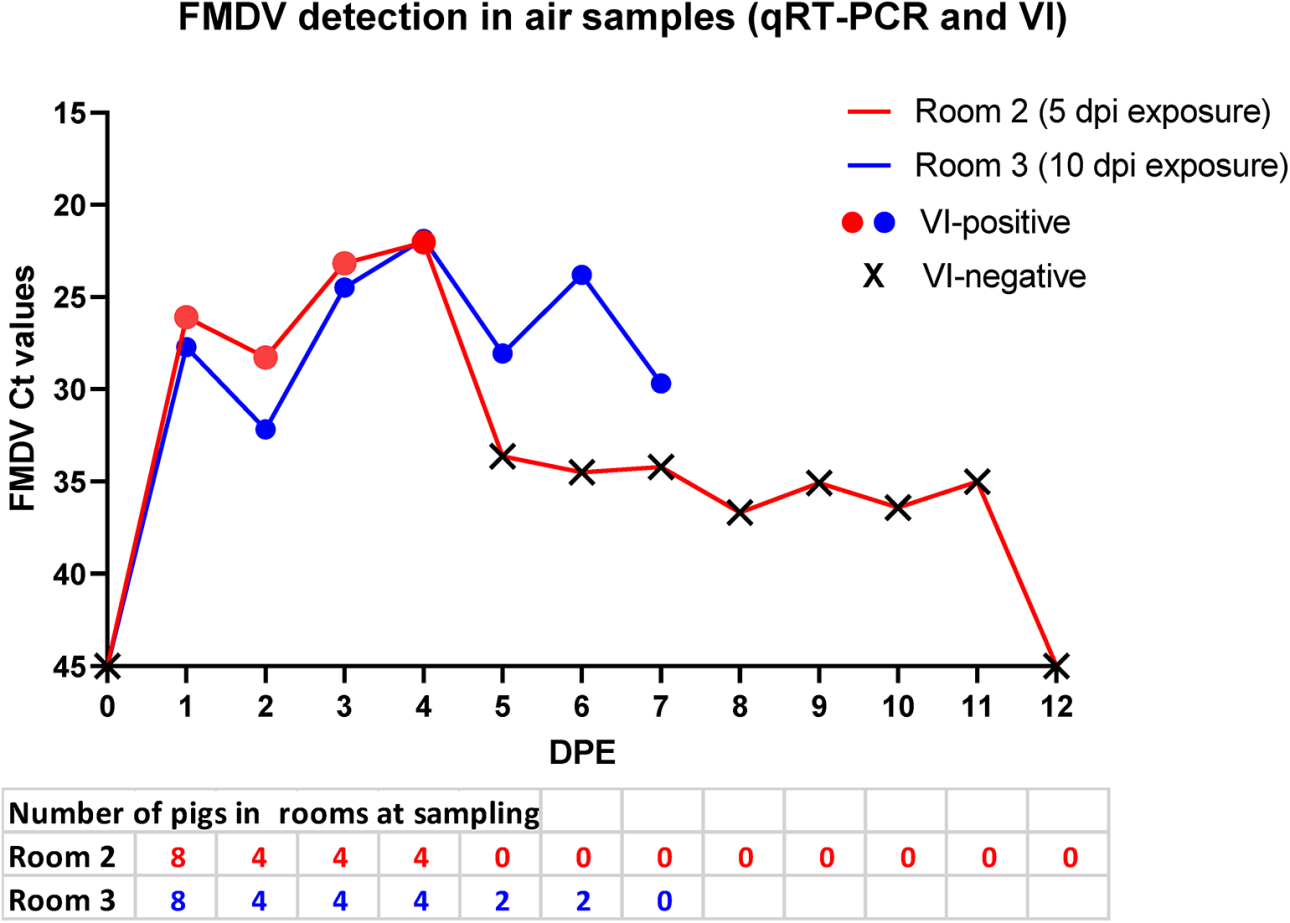
FMDV detection in air samples. FMDV RNA quantities and isolation of infectious FMDV from dry air filters collected from isolation rooms housing pigs that were exposed to FMDV-infected donor pigs for 24 h at 5 (red) or 10 (blue) days post infection of the donors. The Y-axis represents FMDV RNA quantities (Ct values) determined by qRT-PCR. Colored symbols indicate samples from which infectious virus was isolated, while X indicate virus isolation-negative samples. The donor pigs were in the sampled rooms from 0 to 1 days post exposure (dpe). The 4 contact-exposed pigs in group 2 (red) were euthanized and removed at 4 dpe. Two of 4 pigs in room 3 (blue) were euthanized at 4 dpe, and the remaining 2 pigs at 6 dpe. Air sampling continued for 1 or 8 days after removal of the last pig from the rooms.

Air sampling was performed in the rooms housing study groups 2 and 3, starting at 1 day prior to contact exposure, and ending at 8 or 1 day(s) after removal of the pigs from the rooms for group 2 and 3, respectively (Figure 2). The inoculated donor pigs were housed with the contact groups from 0 to 1 dpe. In both rooms, detected FMDV RNA levels decreased slightly after removal of the donor pigs (2 dpe), to then increase by the following day (3dpe), suggesting increased virus shedding by the newly infected contact-exposed pigs. The 4 pigs in group 2 were euthanized at 4 dpe, after which the room was rinsed with steam - hot water hosing with no detergent or disinfectant. Low levels of FMDV RNA was detected in air samples from this room for another 7 days (up to 11 dpe), but there were no VI-positive samples after removal of the pigs (Figure 2). Two pigs from group 3 were euthanized at 5 dpe, and the other 2 at 6 dpe. The room was similarly washed after removal of the pigs, and the final air filter sample was collected the following day. All air filter samples from room 3 were positive by both qRT-PCR and VI (Figure 2).

### Post-mortem Viability of FMDV in Skeletal Muscle and Vesicular Epithelium

Post-mortem sampling of vesicle epithelium and skeletal muscle was performed on 4 distinct batches of pig carcasses that had been infected with either FMDV A24 Cruzeiro or FMDV O/SKR/2010, following slightly different sampling schedules.

**Figure 3:**
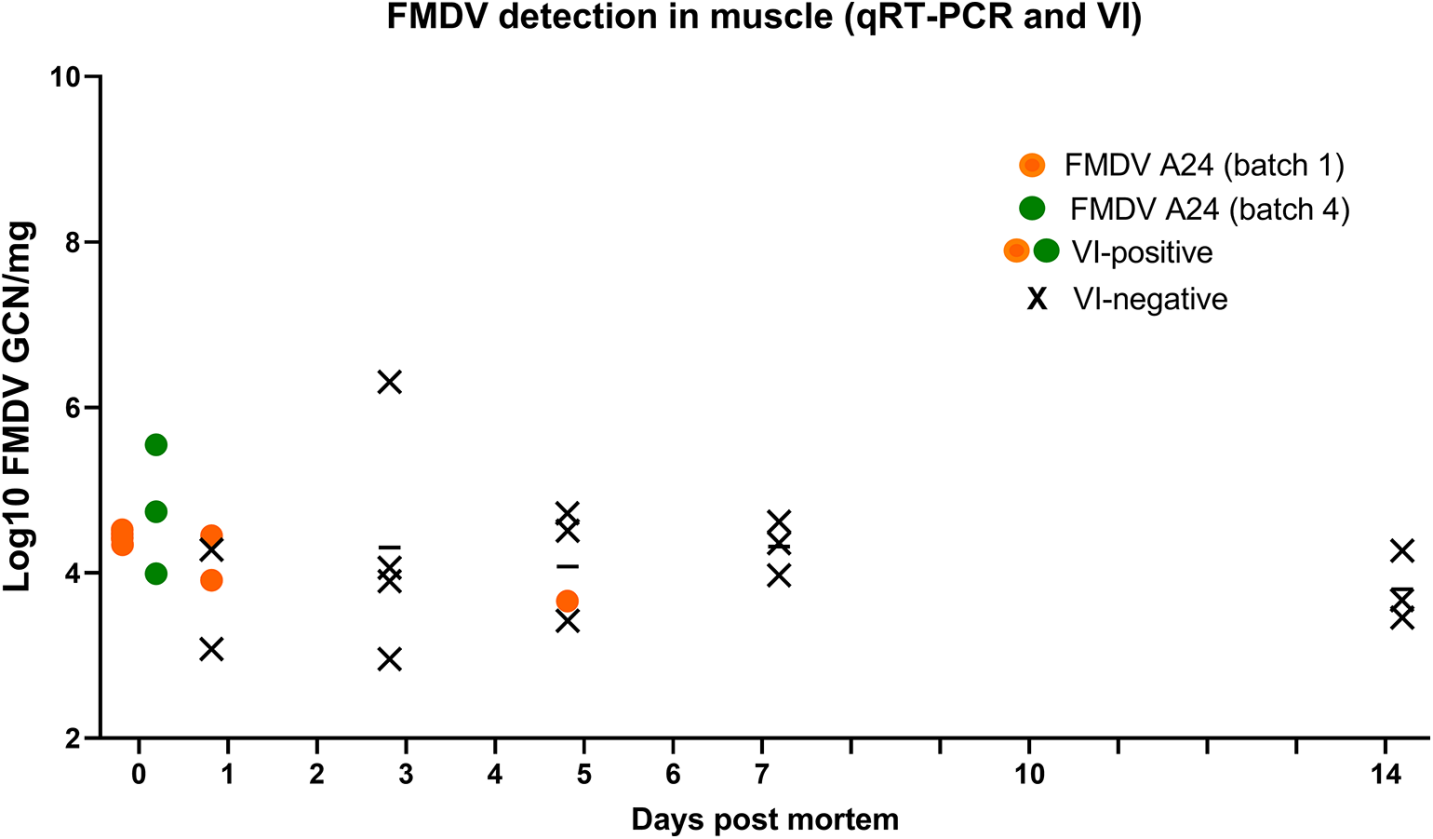
FMDV detection in the semitendinosus muscle of pig carcasses. FMDV RNA quantities (log_10_ GCN/mg) in muscle samples from FMDV-infected pigs obtained from 0 to 14 days post mortem (dpm) from carcasses stored at 4^o^C. Colored symbols indicate samples from which infectious virus was isolated, while X indicate virus isolation-negative samples. Carcasses from batch 1 (*n* = 4) were samples at 0, 1, 3, and 5 dpm and carcasses from batch 2 (*n* = 3) were sampled at 0, 7, and 14 dpm. All symbols represent individual sample replicates (*n* = 7), horizontal lines are means.

Samples of semitendinosus muscle were obtained from batches 1 and 4 (*n* = 7), which had both been infected with FMDV A24 Cruzeiro. Low to moderate quantities of FMDV RNA (3.1-6.3 log_10_ GCN/mg) were detected in all muscle samples (Figure 3). However, virus isolation was only positive at 0 dpm (directly post euthanasia), 1 dpm (2 of 4 samples), and 5 dpm (1 of 4 samples; Figure 3).

**Figure 4:**
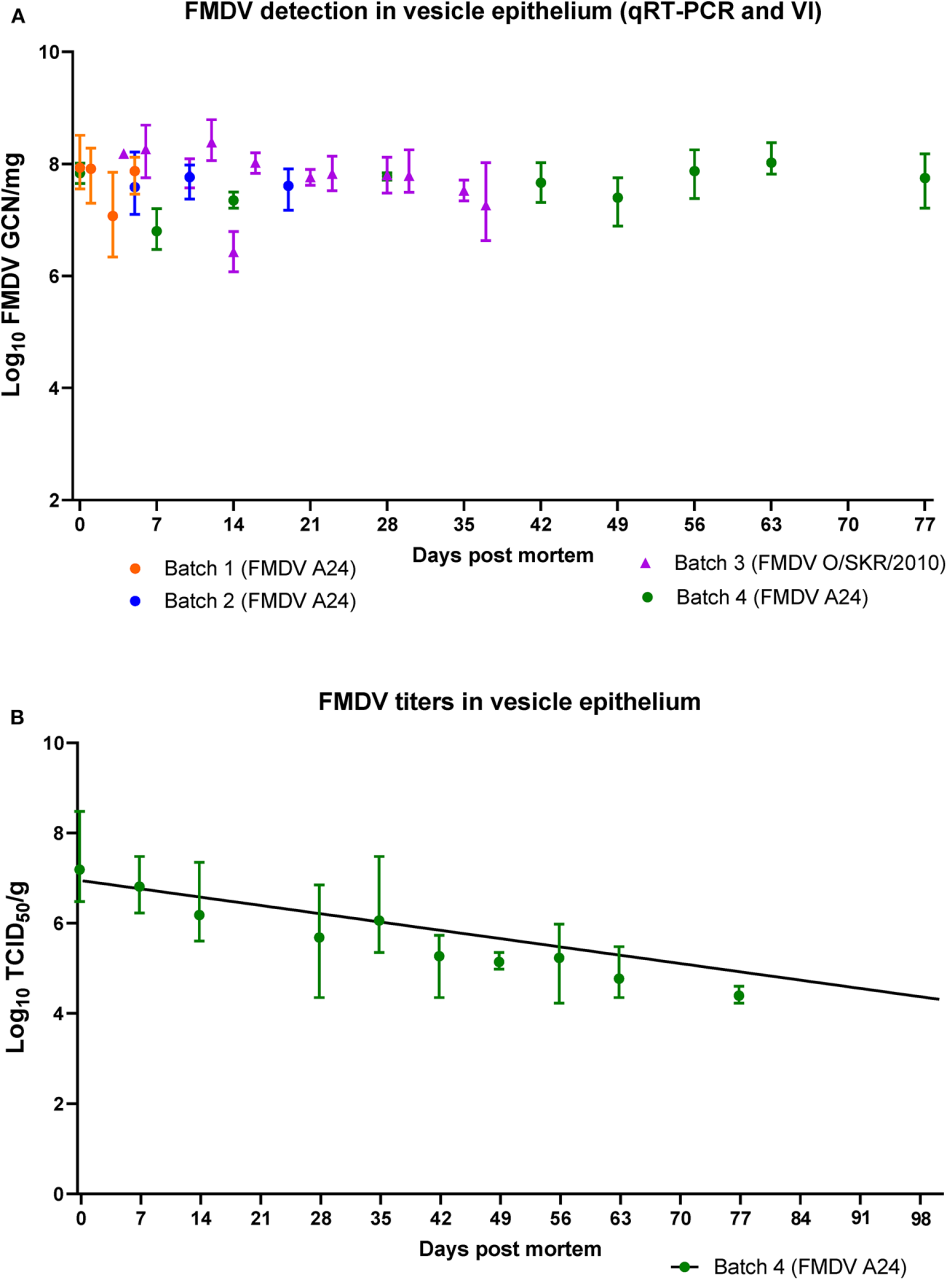
FMDV detection in vesicle epithelium of pig carcasses. **(A)** FMDV quantities (log_10_ GCN/mg) in samples of vesicle epithelium obtained from 4 separate batches of pig carcasses infected with FMDV A24 Cruzeiro or FMDV O/SKR/2010 and stored at 4^o^C from 0 to 77 days post mortem (dpm). All samples were positive by virus isolation. **(B)** FMDV titers (log10 TCID_50_/g) in vesicle epithelium obtained from 3 carcasses infected with FMDV A24 Cruzeiro from 0 to 77 dpm. All plotted values represent geometric means and range. The fitted line represents a simple linear regression with day as the independent variable and individual sample titers as the dependent variable (slope = −0.034, half-life = 128 days).

By contrast, all vesicle epithelium samples obtained from batches 1 through 4 (*n* = 13) from 0 to 77 dpm were positive both by qRT-PCR and VI. FMDV RNA quantities were substantially higher compared to muscle samples (6.34-8.12 log_10_ GCN/mg; Figure 4A). There were no differences in RNA quantities or duration of viability across batches or viruses (the latest samples from FMDV O/SKR/2010-infected carcasses were obtained at 37 dpm).

Virus titrations were performed on all vesicle epithelium samples from batch 4 carcasses (0–77 dpm). Titers ranged from an average of 10^7.2^TCID_50_/g at 0 dpm, to an average of 10^4.4^ TCID_50_/g at 77 dpm (Figure 4B). A simple linear regression fitted to the viral decay data had a negative slope (−0.034). The half-life was estimated to be 128 days and the X-intercept (titer = 10^0^) was estimated to be 203 days (95% confidence interval = 159 to 295 days).

**Figure 5:**
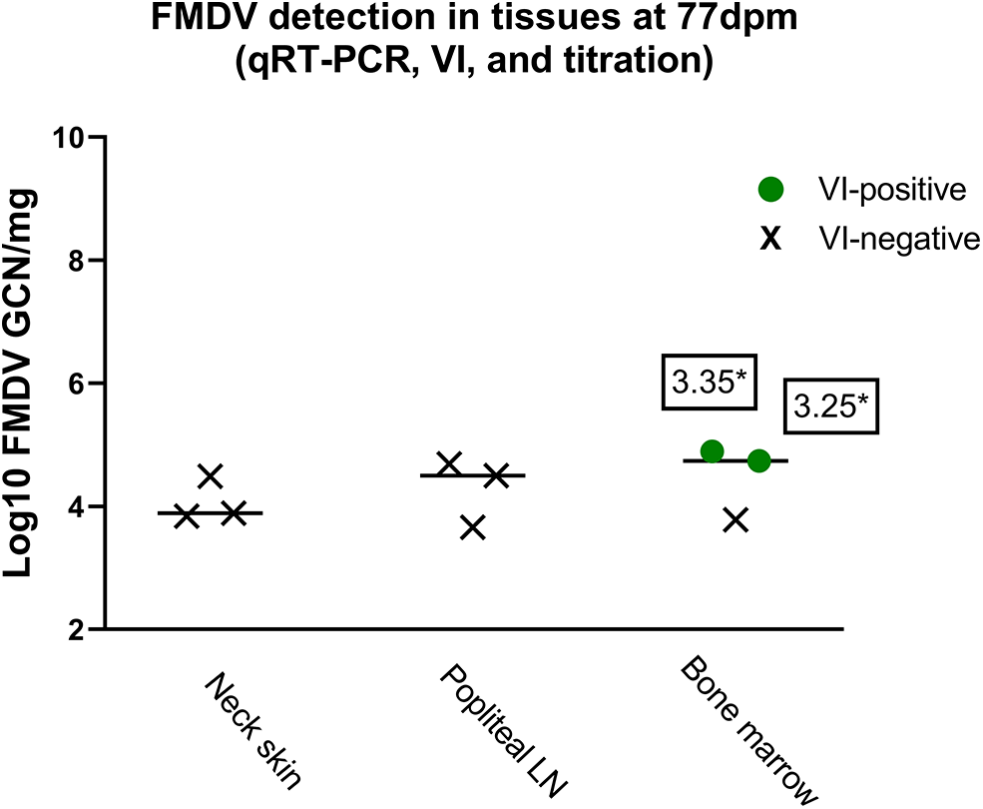
FMDV detection in select tissue samples at 77 days post mortem. FMDV quantities (log_10_ GCN/mg) in samples of submandibular lymph nodes, neck skin, and bone marrow harvested from carcasses of pigs infected with FMDV A24 Cruzeiro after 77 days of storage at 4^o^C. Colored symbols indicate samples from which infectious virus was isolated, while X indicate virus isolation-negative samples. *FMDV titers in two samples of virus isolation-positive bone marrow were 10^3.35^ and 10^3.25^ TCID_50_/g, respectively. All symbols represent individual sample replicates (*n* = 3), horizontal lines are means.

FMDV RNA was detected in submandibular lymph nodes, neck skin, and bone marrow harvested from FMDV A24-infected carcasses at 77 dpm (3.66–4.89 log_10_ GCN/mg; Figure 5). Two bone marrow samples were VI-positive, with titers of 10^3.25^ and 10^3.35^ TCID_50_/g, respectively (Figure 5).

## Discussion

Data-driven models for FMD outbreak simulations represent a critical component of FMD preparedness in countries that are normally free of FMD (6). Although a considerable amount of data describing FMD progression in infected animals can be extrapolated from published experimental studies, only a fraction of such studies were originally designed to evaluate disease transmission. Replacement of actual transmission data by more readily available proxy measures can lead to substantially skewed estimates for infectivity (10, 11). Additionally, as disease transmission is highly context-dependent, it is important that estimates used for model parameterization are appropriate for the host species and housing/management conditions represented in the model. Previous studies have shown that individual housing of animals for one-on-one contact transmission trials can substantially reduce disease transmission (34). Thus, even though a one-to-one study design allows for precise measurements of virus shedding from individual animals for transmission proxy evaluation, the set-up may provide skewed estimates as the transmission characteristics of the disease are altered compared to conventional group-housing settings.

In this current investigation, we addressed a critical knowledge gap related to the end (duration) of infectiousness in group-housed FMDV-infected pigs. A single group of donor pigs subjected to simulated-natural FMDV exposure were used to expose sequential groups of contact pigs at pre-determined time points during late infection. The outcome of this trial demonstrated that transmission occurred at 5 and 10 dpi, but not at 15 dpi. The clinical conditions of the donor pigs improved throughout the study. At 5 dpi, although non-febrile and ambulant, there was still visible lameness and some reluctance to walk within the group. At 10 dpi, the donor pigs were more active, and were freely walking/running during the clinical examination and relocation to the contact exposure room. Although moderate quantities of FMDV RNA were measured in OPF of the donor pigs at 10 dpi, the titers of infectious virus were below the limit of detection in all but 1 donor pig at the start of contact exposure. There were, however, healing FMD lesions at the coronary bands of all the donor pigs, and it is highly possible that the contagion of donor pigs at this time point was associated with virus in residual lesions rather than in aerosols or secretions. This is further supported by the fact that FMDV in secretions would be expected to be associated with secretory immunoglobulin during later stages of infection.

Virus shedding, as defined by detection of FMDV RNA in OPF, was still detectable in samples from the donor pigs at the beginning and end of the last contact exposure at 15–16 dpi. Furthermore, OPF from all the contact-exposed pigs in group 4 was positive for FMDV RNA at the end of the contact exposure. These findings support the concept of a minimum quantity of FMDV being required to cause infection in exposed pigs (35–37), and that the cumulative amount of virus that the pigs in group 4 were exposed to, was below the threshold of infectivity.

We have previously used a similar experimental design to demonstrate that FMDV-infected pigs are capable of transmitting disease ~1 day prior to the appearance of clinical signs (10, 20). During early infection, successful transmission was associated with increasing quantities of FMDV RNA detected in OPF of the donor pigs (20, 37). The findings from this current study suggests that a similar quantitative shedding approach is not appropriate to determine the end of the infectious period. Specifically, the FMDV RNA quantities detected in OPF from the donor pigs were similar between the second exposure at 10-11 dpi and the third exposure at 15–16 dpi despite the transition from contagious to non-contagious status. Based upon this finding, it is likely that the contagion associated with pigs in late phases of FMD is not due to FMDV present in OPF.

The experimental approach of using the same group of donor pigs to expose multiple groups of contact pigs was intended to minimize sources of variation other than the time post infection of the donors. Although this design may have resulted in additional stress of the donor pigs as they were moved between the contact groups, we believe this would not have affected experimental transmission since contagion was not correlated with shedding of FMDV in donors. Rather, the remnant vesicle epithelium on the donor pigs contained high quantities of infectious virus, and this is likely to have been the most substantial determinant of transmission during late stages of infection.

The combined output from the current and previous transmission studies suggests that the infectious period for FMDV-infected pigs lasts for at least 9 days (1 through 10 dpi), which is substantially longer than what has been published for cattle (11). Furthermore, this suggests that the rate of propagation of an FMD outbreak may be substantially impacted by the livestock composition within the affected region.

Nonetheless, it is noteworthy that the cumulative evidence from direct transmission experiments herein and previously published (20, 30) indicate that the actual infectious period in FMDV-infected pigs is shorter than what would have been estimated if using detection of FMDV RNA in OPF as a proxy for infectiousness.

The transmission study reported herein was performed using a single FMDV strain and limited animal numbers. It would thus be relevant to expand the data set by performing similar investigations using additional FMDV strains since previous experimental studies have suggested that there may be strain-specific variability in FMDV transmission parameters (21) and environmental stability.

FMDV virulence and host tropism are known to vary by strain rather than serotype as is demonstrated by the FMDV O Cathay lineage which differs from other serotype O FMDVs by being highly infectious to pigs, but not cattle (38, 39). Therefore, in order to generate robust and representative data, experimental determinations of end-of-infectiousness should ideally be performed for multiple strains within relevant serotypes.

We have previously shown that infectious FMDV could be detected in ambient air for up to 6 days after removal of infected pigs from a room that housed vaccinated (clinically protected) cattle (40), whereas no virus was detected in rooms housing similarly vaccinated cattle that had been infected by intra-nasopharyngeal deposition of the same virus (unpublished). In this current investigation, isolation of virus from air filters was only possible for up to 24 h after removal of infected pigs, while detection of FMDV RNA continued for up to 7 days. It is likely that the presence of cattle in the room of the previous study had a substantial impact on the humidity and temperature in the room, possibly affecting environmental survival of the virus. Additional environmental sampling, such as swabbing of dry surfaces, would be needed to better assess the residual contagion in mechanically cleaned spaces.

FMDV is known to be sensitive to high temperatures and pH values outside a range of 7–7.5 (41–43). It has also been shown that FMDV in muscle tissue gets inactivated within few days post mortem, even when stored at low temperatures (18), which was consistent with the findings from the current study. This is presumed to be associated with the generation of lactic acid from glycogen due to autolysis in muscle. In the current study, the post-mortem viability of FMDV in vesicle epithelium was substantially different, as virus isolation from lesion sites was positive, with measurable virus titers, as far out as 77 days post mortem. Due to biosafety regulations, the carcasses used to evaluate FMDV postmortem viability in the current study were stored in a designated cold storage room at 4^o^C, and we were not able to evaluate virus viability at higher temperatures. However, this temperature would realistically simulate an FMD outbreak occurring during the colder season of the year in temperate climate zones. As an example of that phenomenon, it was reported that cold weather substantially impeded decontamination efforts and contributed to dissemination of the November 2010 FMDV serotype O outbreak in South Korea (44).

There is limited published information regarding viability of FMDV in animal products other than meat through prolonged post-mortem durations. One available review paper by Cottral (18) summarizes the data available at that time, and reports that FMDV remained viable in bone marrow stored at 1–4^o^C for up to 7 months, while virus could be isolated from lesions from guinea pigs for as long as 2 years (18). Conversely, more recent studies of alterations of temperature and pH in animal carcasses in warm climatic conditions suggest that FMDV would likely become inactivated within 24 h when ambient temperatures reach 30–35^o^C (45). Additional data is needed to evaluate FMDV viability in vesicle epithelium at varying ranges of temperature and humidity. In this current study, carcasses were kept intact in order to assess if autolysis, putrefaction, and associated acidification occurring within tissues and body cavities would affect conditions at peripheral lesion sites. Based upon the findings of long-term viability of FMDV in porcine tissues, further studies should be performed assessing viability of additional viral strains with longer duration and variations in ambient conditions.

## Conclusions

The current investigation demonstrated that FMDV A24-infected pigs were capable of transmitting disease as late as 10 days post infection, which corresponded to 9 days after presumed onset of infectivity (20), and 8 days after appearance of clinical signs. The same pigs were non-contagious at 15 dpi. There is clearly some uncertainty associated with this range as there were no contact trials performed between 10 and 15 dpi. However, one important detail to note is that pigs that were largely clinically recovered, with only minor remnants of FMD lesions, were still capable of transmitting disease. Airborne contagion diminished shortly after removal of infected animals and mechanical cleaning of isolation rooms. However, evidence from previous studies suggest that this may vary substantially, likely depending on relative humidity. Although FMDV in muscle was inactivated within few days post mortem, contagion in vesicle epithelium from intact carcasses stored at 4^o^C remained high through 11 weeks. This finding should be considered in relation to appropriate handling and disposal of animal carcasses during FMD outbreaks, especially in cold weather conditions. describing the end of infectiousness in pigs and FMDV viability in post-mortem porcine tissues update the available parameters for modeling of FMD in populations of pigs.

## Data Availability Statement

The raw data supporting the conclusions of this article will be made available by the authors, without undue reservation, to any qualified researcher.

## Ethics Statement

The animal study was reviewed and approved by Plum Island Animal Disease Center Institutional Animal Care and Use Committee.

## Author Contributions

CS coordinated and executed the animal experiments and drafted the manuscript. MB contributed to study design, execution of the animal experiments, and interpretation of results. GS and EH performed sample analyses and interpretation of data. JA, AD, and CS conceived, coordinated and oversaw the work. All authors have critically reviewed and revised the manuscript and approved the final product.

## Conflict of Interest

The authors declare that the research was conducted in the absence of any commercial or financial relationships that could be construed as a potential conflict of interest.

## References

[1] Grubman MJ Baxt B. Foot-and-mouth disease. Clin Microbiol Rev. (2004) 17:465–93. 10.1128/CMR.17.2.465-493.2004 15084510 PMC387408

[2] Arzt J Baxt B Grubman MJ Jackson T Juleff N Rhyan J. The pathogenesis of foot-and-mouth disease II: viral pathways in swine, small ruminants, and wildlife; myotropism, chronic syndromes, and molecular virus-host interactions. Transbound Emerg Dis. (2011) 58:305–26. 10.1111/j.1865-1682.2011.01236.x 21672184

[3] Arzt J Juleff N Zhang Z Rodriguez LL The pathogenesis of foot-and-mouth disease i: viral pathways in cattle. Transbound Emerg Dis. (2011) 58:291–304. 10.1111/j.1865-1682.2011.01204.x 21366894

[4] Knight-Jones TJ Robinson L Charleston B Rodriguez LL Gay CG Sumption KJ. Global foot-and-Mouth disease research update and gap analysis: 1–overview of global status and research needs. Transbound Emerg Dis. (2016) 63 Suppl 1:3–13. 10.1111/tbed.12528 27320162

[5] Knight-Jones TJ Rushton J. The economic impacts of foot and mouth disease - what are they, how big are they and where do they occur? Prev Vet Med. (2013) 112:161–73. 10.1016/j.prevetmed.2013.07.013 23958457 PMC3989032

[6] Pomeroy LW Bansal S Tildesley M Moreno-Torres KI Moritz M Xiao N. Data-Driven models of foot-and-Mouth disease dynamics: a Review. Transbound Emerg Dis. (2017) 64:716–28. 10.1111/tbed.12437 26576514 PMC5205574

[7] Kinsley AC Patterson G VanderWaal KL Craft ME Perez AM. Parameter values for epidemiological models of foot-and-Mouth disease in swine. Front Vet Sci. (2016) 3:44. 10.3389/fvets.2016.00044 27314002 PMC4887472

[8] Mardones F Perez A Sanchez J Alkhamis M Carpenter T. Parameterization of the duration of infection stages of serotype o foot-and-mouth disease virus: an analytical review and meta-analysis with application to simulation models. Vet Res. (2010) 41:45. 10.1051/vetres/2010017 20205988 PMC2850150

[9] Yadav S Stenfeldt C Branan MA Moreno-Torres KI Holmstrom LK Delgado AH. Parameterization of the durations of phases of foot-And-mouth disease in cattle. Front Vet Sci. (2019) 6:263. 10.3389/fvets.2019.00263 31448297 PMC6696987

[10] Arzt J Branan MA Delgado AH Yadav S Moreno-Torres KI Tildesley MJ. Quantitative impacts of incubation phase transmission of foot-and-mouth disease virus. Sci Rep. (2019) 9:2707. 10.1038/s41598-019-39029-0 30804426 PMC6389902

[11] Charleston B Bankowski BM Gubbins S Chase-Topping ME Schley D Howey R. Relationship between clinical signs and transmission of an infectious disease and the implications for control. Science. (2011) 332:726–9. 10.1126/science.1199884 21551063 PMC5844461

[12] Walz E Evanson J Sampedro F VanderWaal K Goldsmith T. Planning “Plan b”: the case of moving cattle from an infected feedlot premises during a hypothetical widespread fMD outbreak in the united states. Front Vet Sci. (2020) 6:484. 10.3389/fvets.2019.00484 31998764 PMC6964524

[13] de Klerk PF. Carcass disposal: lessons from the netherlands after the foot and mouth disease outbreak of 2001. Rev Sci Tech. (2002) 21:789–96. 10.20506/rst.21.3.1376 12523715

[14] Scudamore JM Trevelyan GM Tas MV Varley EM Hickman GAW. Carcass disposal: lessons from great britain following the foot and mouth disease outbreaks of 2001. Rev Sci Tech. (2002) 21:775–87. 10.20506/rst.21.3.1377 12523714

[15] Walz E Middleton J Sampedro F VanderWaal K Malladi S Goldsmith T. Modeling the transmission of foot and mouth disease to inform transportation of infected carcasses to a disposal site during an outbreak event. Front Vet Sci. (2020) 6:501. 10.3389/fvets.2019.00501 31993448 PMC6971117

[16] Paton DJ Sinclair M Rodríguez R. Qualitative assessment of the commodity risk for spread of foot-and-Mouth disease associated with international trade in deboned beef. Trans Emerg Dis. (2010) 57:115–34. 10.1111/j.1865-1682.2010.01137.x 20569417

[17] Greiner N Jensen TB FMDV Survival in Meat. Required Input From a Risk Assessment Point of View. Rome: Food and Agriculture Organization (2005).

[18] Cottral GE. Persistence of foot-and-mouth disease virus in animals, their products and the environment. Bull Off Int Epizoot. (1969) 71:549–68. 4324721

[19] Stenfeldt C Pacheco JM Rodriguez LL Arzt J. Early events in the pathogenesis of foot-and-mouth disease in pigs; identification of oropharyngeal tonsils as sites of primary and sustained viral replication. PLoS ONE. (2014) 9:e106859. 10.1371/journal.pone.0106859 25184288 PMC4153717

[20] Stenfeldt C Pacheco JM Brito BP Moreno-Torres KI Branan MA Delgado AH. Transmission of foot-and-Mouth disease virus during the incubation period in pigs. Front Vet Sci. (2016) 3:105. 10.3389/fvets.2016.00105 27917386 PMC5116750

[21] Pacheco JM Tucker M Hartwig E Bishop E Arzt J Rodriguez L. Direct contact transmission of three different foot-and-mouth disease virus strains in swine demonstrates important strain-specific differences. Vet J. (2012) 193:456–63. 10.1016/j.tvjl.2012.01.012 22342891

[22] Stenfeldt C Pacheco JM Rodriguez LL Arzt J. Infection dynamics of foot-and-mouth disease virus in pigs using two novel simulated-natural inoculation methods. Res Vet Sci. (2014) 96:396–405. 10.1016/j.rvsc.2014.01.009 24548596

[23] Pacheco JM Mason PW. Evaluation of infectivity and transmission of different asian foot-and-mouth disease viruses in swine. J Vet Sci. (2010) 11:133–42. 10.4142/jvs.2010.11.2.133 20458154 PMC2873813

[24] LaRocco M Krug PW Kramer E Ahmed Z Pacheco JM Duque H. A continuous bovine kidney cell line constitutively expressing bovine alphavbeta6 integrin has increased susceptibility to foot-and-mouth disease virus. J Clin Microbiol. (2013) 51:1714–20. 10.1128/JCM.03370-12 23515553 PMC3716081

[25] LaRocco M Krug PW Kramer E Ahmed Z Pacheco JM Duque H. A continuous bovine kidney cell line constitutively expressing bovine alphaVbeta6 integrin has increased susceptibility to foot-and-mouth disease virus. J Clin Microbiol. (2015). 53:755. 10.1128/JCM.03220-14 25617444 PMC4298512

[26] Pacheco JM Henry TM O'Donnell VK Gregory JB Mason PW. Role of nonstructural proteins 3A and 3B in host range and pathogenicity of foot-and-mouth disease virus. J Virol. (2003) 77:13017–27. 10.1128/JVI.77.24.13017-13027.2003 14645558 PMC296074

[27] Pacheco JM Lee KN Eschbaumer M Bishop EA Hartwig EJ Pauszek SJ. Evaluation of infectivity, virulence and transmission of fDMV field strains of serotypes o and a Isolated in 2010 from outbreaks in the republic of korea. PLoS ONE. (2016) 11:e0146445. 10.1371/journal.pone.0146445 26735130 PMC4703371

[28] Callahan JD Brown F Osorio FA Sur JH Kramer E Long GW. Use of a portable real-time reverse transcriptase-polymerase chain reaction assay for rapid detection of foot-and-mouth disease virus. J Am Vet Med Assoc. (2002) 220:1636–42. 10.2460/javma.2002.220.1636 12051502

[29] Rasmussen TB Uttenthal A de Stricker K Belak S Storgaard T. Development of a novel quantitative real-time RT-PCR assay for the simultaneous detection of all serotypes of foot-and-mouth disease virus. Arch Virol. (2003) 148:2005–21. 10.1007/s00705-003-0145-2 14551821

[30] Stenfeldt C Pacheco JM Smoliga G Bishop E Pauszek S Hartwig E. Detection of foot-and-mouth disease virus RNA and capsid protein in lymphoid tissues of convalescent pigs does not indicate existence of a carrier state. Transbound Emerg Dis. (2016) 63:152–64. 10.1111/tbed.12235 24943477

[31] Swaney LMA. continuous bovine kidney cell line for routine assays of foot-and-mouth disease virus. Vet Microbiol. (1988) 18:1–14. 10.1016/0378-1135(88)90111-3 2847400

[32] Pacheco JM Arzt J Rodriguez LL. Early events in the pathogenesis of foot-and-mouth disease in cattle after controlled aerosol exposure. Vet J. (2010) 183:46–53. 10.1016/j.tvjl.2008.08.023 18930417

[33] Reed LJ Muench H. A simple method of estimating fifty percent endpoints. Am J Hygiene. (1938) 27:493–7. 10.1093/oxfordjournals.aje.a11840815955576

[34] Quan M Murphy CM Zhang Z Durand S Esteves I Doel C. Influence of exposure intensity on the efficiency and speed of transmission of foot-and-mouth disease. J Comp Pathol. (2009) 140:225–37. 10.1016/j.jcpa.2008.12.002 19215941

[35] Sellers RF Quantitative aspects of the spread of foot and mouth disease. Vet Bull. (1971) 41:431–9.

[36] Arzt J Belsham GJ Lohse L Botner A Stenfeldt C. Transmission of foot-and-Mouth disease from persistently infected carrier cattle to naive cattle via transfer of oropharyngeal fluid. mSphere. (2018) 3:e00365–18. 10.1128/mSphere.00365-18 30209130 PMC6135961

[37] Moreno-Torres KI Brito BP Branan MA Rodriguez LL Delgado AH Stenfeldt C. Foot-and-Mouth disease infection dynamics in contact-Exposed pigs are determined by the estimated exposure dose. Front Vet Sci. (2018) 5:167. 10.3389/fvets.2018.00167 30079340 PMC6062637

[38] Yang PC Chu RM Chung WB Sung HT. Epidemiological characteristics and financial costs of the 1997 foot-and-mouth disease epidemic in taiwan. Vet Rec. (1999) 145:731–4. 10.1136/vr.145.25.731 10972111

[39] Dunn CS Donaldson AI. Natural adaption to pigs of a taiwanese isolate of foot-and-mouth disease virus. Vet Rec. (1997) 141:174–5. 10.1136/vr.141.7.174 9290197

[40] Stenfeldt C Hartwig EJ Smoliga GR Palinski R Silva EB Bertram MR. Contact challenge of cattle with foot-and-Mouth disease virus validates the role of the nasopharyngeal epithelium as the site of primary and persistent infection. mSphere. (2018) 3:18. 10.1128/mSphere.00493-18 30541776 PMC6291620

[41] Scott KA Maake L Botha E Theron J Maree FF. Inherent biophysical stability of foot-and-mouth disease sAT1, sAT2 and sAT3 viruses. Virus Res. (2019) 264:45–55. 10.1016/j.virusres.2019.02.012 30807778

[42] Bachrach HL Breese SS Jr Callis JJ Hess WR Patty RE. Inactivation of foot-and-mouth disease virus by pH and temperature changes and by formaldehyde. Proc Soc Exp Biol Med. (1957) 95:147–52. 10.3181/00379727-95-23148 13432017

[43] Caridi F Vazquez-Calvo A Sobrino F Martin-Acebes MA. The pH stability of foot-and-Mouth disease virus particles is modulated by residues located at the pentameric interface and in the n Terminus of vP1. J Virol. (2015) 89:5633–42. 10.1128/JVI.03358-14 25762735 PMC4442502

[44] Yoon H Yoon SS Kim YJ Moon OK Wee SH Joo YS. Epidemiology of the foot-and-mouth disease serotype o epidemic of november 2010 to april 2011 in the republic of korea. Transbound Emerg Dis. (2013) 62:252–63. 10.1111/tbed.12109 23731597

[45] Hunnam J Duff K Wingett M Brayley E Williamson G. Effect of carcase decomposition on the inactivation of foot-and-mouth disease virus under northern australian conditions. Aust Vet J. (2018) 96:332–40. 10.1111/avj.12731 30152065

